# COVID-19 symptom relationship to antibody response and ACE2 neutralization in recovered health systems employees before and after mRNA BNT162b2 COVID-19 vaccine

**DOI:** 10.1371/journal.pone.0273323

**Published:** 2022-09-09

**Authors:** Gregory Huhn, Josh Poorbaugh, Lin Zhang, Stephanie Beasley, Ajay Nirula, Jennifer Brothers, Sharon Welbel, James Wilson, Sheena Gillani, Kathleen M. Weber, Ralph Morack, Kody Keckler, Robert J. Benschop

**Affiliations:** 1 The Ruth M. Rothstein CORE Center, Cook County Health, Chicago, Illinois, United States of America; 2 Rush University Medical Center, Chicago, Illinois, United States of America; 3 Eli Lilly and Company, Indianapolis, Indiana, United States of America; 4 Hektoen Institute of Medicine, Chicago, Illinois, United States of America; Translational Health Science & Technology Institute, INDIA

## Abstract

**Background:**

The humoral response to SARS-CoV-2 can provide immunity and prevent reinfection. However, less is known about how the diversity, magnitude, and length of the antibody response after a primary infection is associated with symptoms, post-infection immunity, and post-vaccinated immunity.

**Methods:**

Cook County Health employees provided blood samples and completed an online survey 8–10 weeks after a PCR-confirmed positive SARS-CoV-2 test (pre-vaccinated, N = 41) and again, 1–4 weeks after completion of a 2-dose series mRNA BNT162b2 COVID-19 vaccine (post-vaccinated, N = 27). Associations were evaluated between SARS-CoV-2 antibody titers, participant demographics, and clinical characteristics. Antibody titers and angiotensin-converting enzyme 2 (ACE2) neutralization were compared before and after the mRNA BNT162b2 COVID-19 vaccine.

**Results:**

Antibody titers to the spike protein (ST4), receptor binding domain (RBD), and RBD mutant D614G were significantly associated with anosmia and ageusia, cough, and fever. Spike protein antibody titers and ACE2 neutralization were significantly higher in participants that presented with these symptoms. Antibody titers to the spike protein N-terminal domain (NTD), RBD, and ST4, and ACE2 IC50 were significantly higher in all post-vaccinated participant samples compared to pre-vaccinated participant sample, and not dependent on previously reported symptoms.

**Conclusions:**

Spike protein antibody titers and ACE2 neutralization are associated with the presentation of anosmia and ageusia, cough, and fever after SARS-CoV-2 infection. Symptom response to previous SARS-CoV-2 infection did not influence the antibody response from subsequent vaccination. These results suggest a relationship between infection severity and the magnitude of the immune response and provide meaningful insights into COVID-19 immunity according to discrete symptom presentation.

## Introduction

SARS-CoV-2 is a contagious and rapidly mutating virus that produces COVID-19 disease in humans and has consequently resulted in a global pandemic. The fatality ratio of COVID-19 disease increases with specific risk factors, such as age and certain underlying medical conditions, and has resulted in a global death count of over 5.5 million as of January 2022 [1]. Immunity to SARS-CoV-2 infection and reinfection remains the primary objective for disease and mortality control [2].

Immunity can be conferred through the cellular immune response and development of SARS-CoV-2 neutralizing immunoglobulin IgG antibodies [3]. Neutralizing antibodies target epitopes within the full length spike protein or spike protein regions, such as the receptor binding domain (RBD) [4] and N-terminal domain (NTD) [5], preventing viral attachment and entry into angiotensin-converting enzyme 2 (ACE2)-positive cells in an infected host [6]. Antibodies can also develop against epitopes outside of the spike protein, such as at the nucleocapsid protein (NCP) and aid in the overall ability to control the virus [7, 8].

Antibodies to the spike protein and NCP induced by SARS-CoV-2 can result in post-infection immunity up to 8 months post-symptom onset, though variability exists in antibody binding affinity and duration of neutralizing activity [9]. Variability in antibody binding and neutralization of SARS-CoV-2 presents a challenge in predicting duration of immunity to SARS-CoV-2. However, specific symptoms and severity of COVID-19 disease may be predictive of sustained immunity [10] and suggests clinically beneficial relationships to investigate.

The purpose of this study was to identify relationships between the symptoms from SARS-CoV-2 infection and the resulting antibody response. The objectives of this study were to (1) assess antibody titers in plasma samples from health systems employees, 8–10 weeks after onset of COVID-19 disease symptoms, (2) examine relationships among antibody profiles 8–10 weeks after onset of COVID-19 symptoms and demographics, pre-existing medical conditions, disease severity and duration, and symptom inventory and treatment, and (3) compare antibody profiles and ACE2 neutralization before and after a 2-dose series mRNA BNT162b2 COVID-19 vaccine. Investigators hypothesized that spike protein antibody titers would be associated with disease severity and symptoms, and that both spike protein antibody titers and ACE2 neutralization would increase after mRNA BNT162b2 COVID-19 vaccination.

## Methods

### Participant screening, recruitment, and enrollment

Forty-one employees from Cook County Health who tested positive for SARS-CoV-2 with symptomatic illness were enrolled in the study between May 2020 and July 2020. SARS-CoV-2 infection was confirmed through positive detection of SARS-CoV-2 nucleic acids by RT-PCR of nasal swab samples. Phase 1 of the study enrolled participants from May 2020-July 2020, 8–10 weeks after a positive SARS-CoV-2 PCR test, but prior to vaccination (designated pre-vaccinated). Participants were required to provide a 40ml blood sample and complete an online survey with questions related to participant demographics, medical conditions, disease severity and duration, and symptom inventory and treatment (S1 File).

Of 41 participants, 27 (66%) received the 2-dose series mRNA BNT162b2 COVID-19 vaccine 212 days (median, first shot) and 232 days (median, second shot) after the phase 1 visit and blood draw, and subsequently returned for a phase 2 second visit from February 2021-May 2021,1–4 weeks after their second mRNA BNT162b2 COVID-19 vaccine shot (designated post-vaccinated) (S1 Fig). Vaccines were administered at Cook County Hospital following fact sheet guidelines for the mRNA BNT162b2 COVID-19 vaccine (30 μg, Pfizer) [11]. Vaccines were not administered by the investigators of this study, but vaccine records were confirmed by the investigators. The phase 2 visit consisted of a 40ml blood sample and completion of a second online survey with questions related to participant comorbidities and vaccine symptom inventory (S2 File).

Both phases of the study were approved by the Cook County Health Institutional Review Board. Participants provided informed consent and HIPPA authorization prior to study initiation.

### Convalescent plasma collection

40ml of participant blood was collected from an arm vein and transported at room temperature on the same day to the Hektoen Institute of Medicine for phase 1 and the CORE Center Research Lab for phase 2. Plasma was isolated as described previously [12]. Plasma aliquots (1ml) were stored at -80° C and sent to Eli Lilly and Company, Indianapolis for antibody screening.

### Luminex antibody screening

Antibody titers and ACE2-RBD binding inhibition were determined using a custom bead-based immunoassay using Luminex multiplex technology. Luminex xMAP technology is an established platform that allows the simultaneous quantitation of multiple analytes in a single reaction using flow-based detection.

Methods were modified from previously described sample preparation and analysis [12]. Luminex beads were prepared with 15 antigens during analysis of phase 1 samples and included Nucleocapsid Protein (NCP), Spike Protein N-terminal Domain (NTD), Full length Spike Protein (ST4), Receptor Binding Domain 1 & 2 (RBD1, RBD2), SARS-CoV-2 RBD mutant protein antigens (D614G, F490S, N460K, E484Q, and Q493R), and Coronavirus Family protein antigens (SARS-CoV-1, HKU1, OC43, NL63, and 229E). The sequence amino acid lengths are described in Zhang et al., 2021 [13]. RBD mutant antigens were primarily selected based on the variant landscape during participant infection that occurred 8–10 weeks prior to the phase 1 blood draw in May 2020-July 2020. Phase 1 samples were analyzed a second time, in the same batch as phase 2 samples, analysis to allow for direct comparison between pre-vaccinated and post-vaccinated participant samples. The second analysis included antigens for NCP, NTD, ST4, RBD1 & 2, and RBD mutant protein antigens d69-70 and E484Q.

Briefly, participant plasma samples were titrated (1:800–1:8.06E9 or 1:20–1:4.3E8) in phosphate buffered saline-high salt solution (PBS-HS; 0.01 M PBS, 1% bovine serum albumin (BSA), 0.02% Tween, 300 mM NaCl). A minimum titer of 1:800 was imputed for phase 1 samples that failed to generate dilution curves. Addition of ACE2 neutralization required changing the starting dilution from 1:800 to 1:20; therefore during the second sample analysis, a minimum titer of 1:20 was imputed for analysis of phase 1 and 2 samples that failed to generate dilution curves. After titration, diluted plasma samples were combined with Luminex MAGPlex beads coupled with individual antigens and a recombinant, labelled RBD-Phycoerythrin (PE) protein and incubated for 60 minutes to allow endogenous antibodies to bind to either the recombinant RBD-PE or to the antigen-coated Luminex beads. The solution was placed on a magnet to collect the MAGPlex beads while the supernatant was transferred to a new plate, combined with ACE2 coated beads, and incubated for 60 minutes for RBD-ACE2 binding inhibition. To determine antibody titer from the same sample, the beads collected by the magnet were washed and incubated for 60 minutes with anti-IgG-PE beads to detect bound antibodies. All beads on both plates were washed and resuspended in PBS-1% BSA and read using a Luminex FlexMAP3D System with xPONENT software. The titer was evaluated from the median fluorescence intensity (MFI). If the maximum signal of a titration curve was less than the cut point, then the titer was imputed as the smallest dilution. ACE2 neutralization was evaluated from RBD-ACE2 binding inhibition, wherefrom the half maximal inhibitory concentration (IC50) was calculated.

### Statistical analysis

As described by Shankar et al. [14], titers were defined as the value interpolated from the dilution curve where assay values crossed the cut point. Titers and associations with SARS-CoV-2 symptoms were tested using nonparametric t-tests and Benjamini-Hochberg adjustment for multiplicity. A nonparametric paired t-test was used to compare titer and ACE2 IC50 values from pre-vaccination samples and post-vaccination samples (due to the high correlations among titers of epitopes, no multiplicity adjustment was applied). To calculate IC50 of data from the ACE2 neutralization component of the plasma assay, a 4-parameter logistic function was used to estimate the absolute IC50 based on 1/dilution factor. If a sample indicated no neutralization, the IC50 was imputed to 1 (20 times the maximum 1/dilution factor).

## Results

### Participant characteristics

Blood samples were obtained from 41 pre-vaccinated participants who had tested positive for SARS-CoV-2 and 27 returning post-vaccinated participants (Table 1, S1 Fig). Participant-reported demographics (age, sex, race, and ethnicity) were notably similar in pre-vaccinated participants compared to post-vaccinated participants. The median time from a positive SARS-CoV-2 test to when blood was collected in pre-vaccinated participants was 59.5 days (50–121), or 8.5 weeks. The median time from a positive SARS-CoV-2 test to when blood was collected in post-vaccinated participants was 317 days (273–407), or 45 weeks. The median time from the second mRNA BNT162b2 COVID-19 vaccine to when blood was collected for post-vaccinated samples was 23 days (7–29), or 3 weeks.

**Table 1:** Participant-reported demographic and clinical characteristics.

Characteristic	Pre-Vaccinated Participants (N = 41)	Post-Vaccinated Participants (N = 27)
**Age, Mean years (range)**	44.8 (27–68)	43.7 (27–68)
**Sex, No. (%)**		
Female	21/41 (51%)*	13/27 (48%)
Male	20/41 (49%)	14/27 (52%)
**Race, No./total (%)**		
White	15/41 (37%)	13/27 (48%)
Black	13/41 (32%)	6/27 (22%)
Asian	12/41 (29%)	8/27 (30%)
Other	1/41 (2%)	-
**Ethnicity, No./total (%)**		
Hispanic	7/41 (17%)	4/27 (15%)
Not Hispanic	34/41 (83%)	23/27 (85%)
**COVID-19 Symptom Severity**		
Asymptomatic	4/41 (10%)	1/27 (4%)
Minor	26/41 (63%)	20/27 (74%)
Major	10/41 (22%)	5/27 (19%)
Catastrophic	1/41 (2%)	1/27 (4%)
**Time Since Positive SARS-CoV-2 Test, Median days (range)** ^&^	59.5 (50–121)	317 (273–407)
**Time Since Last COVID-19 Symptoms, Median days (range)** ^#^	-	317 (262–484)^
**Vaccinated with BNT162b2 mRNA Vaccine, No./total (%)**	0/41 (0%)	27/27 (100%)
**Time After Second Vaccine Dose, Median days (range)**	-	23 (7–29)

*Percentages may not total 100 because of rounding.

^&^1 participant recorded incorrectly and removed from time calculation.

^#^Time since last COVID-19 Symptoms not recorded in Phase 1 survey

^Only N = 22 out of 27 represented. 2 participants did not record a time since last COVID-19 symptom and 3 participants recorded an incorrect year or the same date as their second visit.

Pre-existing medical conditions were reported in 23 participants. Of these, 14 participants reported ≥2 medical conditions, and 4 participants reported ≥3 medical conditions. Reported pre-existing medical conditions included diabetes (N = 5), hypothyroidism (N = 4), gastrointestinal reflux disease (N = 3), hypertension (N = 5), hyperlipidemia (N = 4), arthritis (N = 3), asthma (N = 2), and remission from or active cancer (N = 3), and minimal reports (N = 1) of mast cell disorder, depression, HIV, HSV, allergic rhinitis, pre-diabetes, prior heart valve surgery or pneumonectomy, heart burn, acromegaly, anemia, vitamin D deficiency, sickle cell disease, and sleep apnea.

Participant-reported COVID-19 symptom severity was subjective in the questionnaire and descriptions defining symptom severity was not provided to the participant (S1 File). Participants were able to report their symptoms as asymptomatic, minor, major, and catastrophic. Only one participant reported catastrophic COVID-19 symptoms and experienced prolonged hospitalization on high flow oxygen.

### SARS-CoV-2 antibody titers after COVID-19 disease and impact of participant-reported COVID-19 symptoms

**Fig 1:**
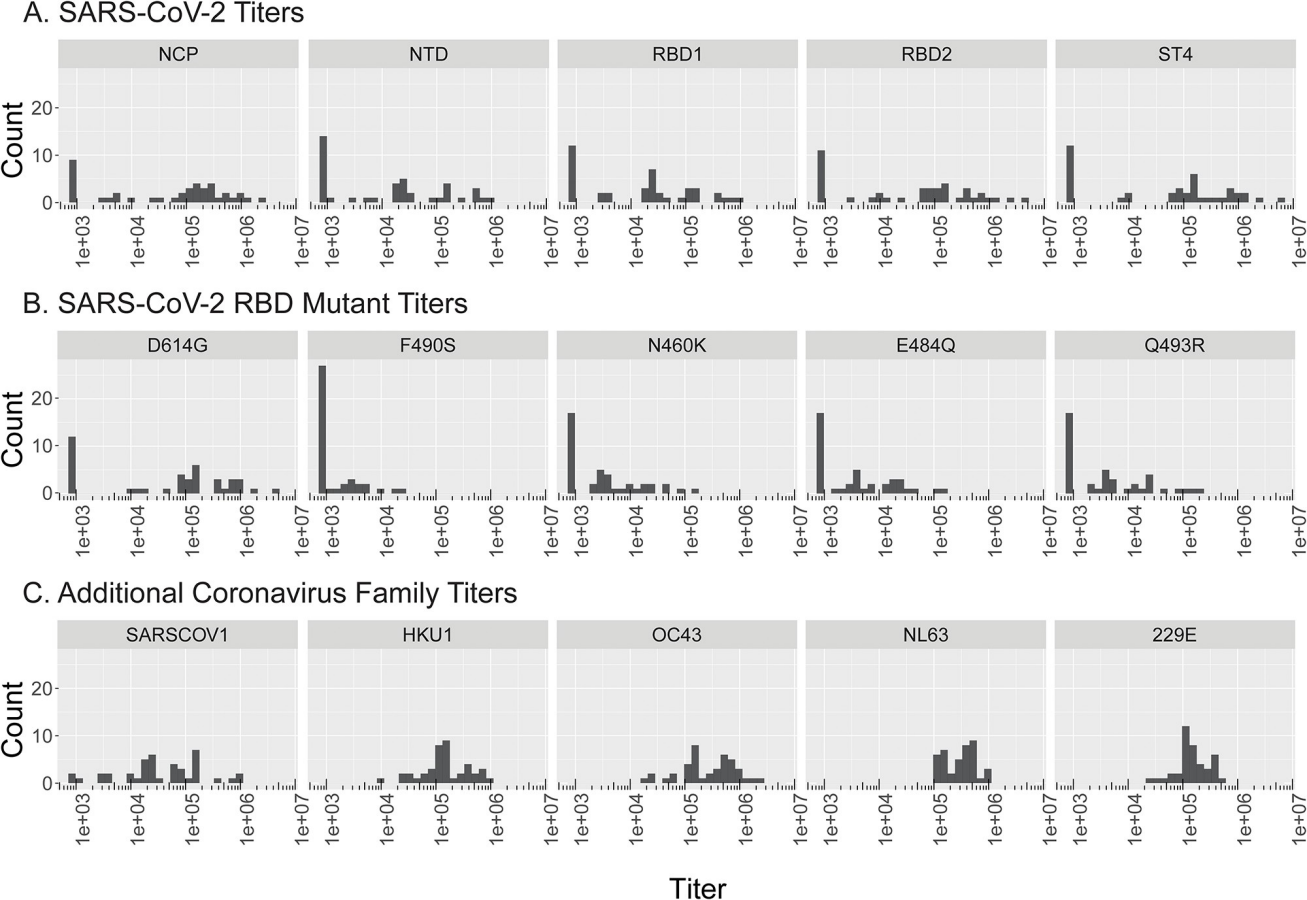
Convalescent plasma SARS-CoV-2, RBD mutant, & coronavirus family antibody titer histograms from pre-vaccinated participants. Convalescent plasma samples from pre-vaccinated participants (n = 41) were analyzed for antibodies against 15 antigens including (A) SARS-CoV-2 spike antigens NCP, NTD, RBD1, RBD2, and ST4, (B) RBD mutant antigens D614G, F490S, N460K, E848Q, Q493R, and (C) Coronavirus family antigens SARS-CoV-1, HKU1, OC43, NL63, and 229E. Histogram of titers for antigens are displayed (x-axis, Titers) against number of participants (y-axis, Count). If the maximum signal of a titration curve is less than the cut point, then the titer is imputed as 800 (smallest dilution).

Plasma isolated from pre-vaccinated participant blood samples were assayed for antigen binding to SARS-CoV-2 proteins and related coronavirus family proteins (Table 2). Titer histograms for SARS-CoV-2, RBD mutants, and coronavirus family proteins overall displayed a wide range of IgG levels (Fig 1). If the maximum signal of a titration curve is less than the cut point, then the titer is imputed as 800 (smallest dilution). Within SARS-CoV-2 proteins, median titer values were highest for NCP (121,840) and ST4 (98,591) (Table 2). SARS-CoV-2 RBD mutant proteins displayed lower median titer values (<3,548), except for D614G (108,678). Proteins from related coronavirus family members (HKU1, OC43, NL63, 229E) consistently displayed high median titer values (>134,673), except for SARS-CoV-1 (27,834).

**Table 2:** Convalescent plasma titers from pre-vaccinated participants.

Antigens	Antigen Abbreviation	N	%Titer = 800	%Titer>800	Median of Titer
**SARS-CoV-2 Proteins**	**NCP**	41	22%	78%	121,840
	**NTD**	41	32%	68%	19,932
	**RBD1**	41	29%	71%	24,696
	**RBD2**	41	27%	73%	71,529
	**ST4**	41	29%	71%	98,591
**SARS-CoV-2 RBD Mutant Proteins**	**D614G**	41	29%	71%	108,678
	**F490S**	41	66%	34%	800
	**N460K**	41	39%	61%	2,655
	**E484Q**	41	41%	59%	2,759
	**Q493R**	41	41%	59%	3,549
**Additional Coronavirus Family Proteins**	**SARS-CoV-1**	41	5%	95%	27,834
	**HKU1**	41	0%	100%	143,098
	**OC43**	41	0%	100%	298,107
	**NL63**	41	0%	100%	333,323
	**229E**	41	0%	100%	134,673

**Fig 2:**
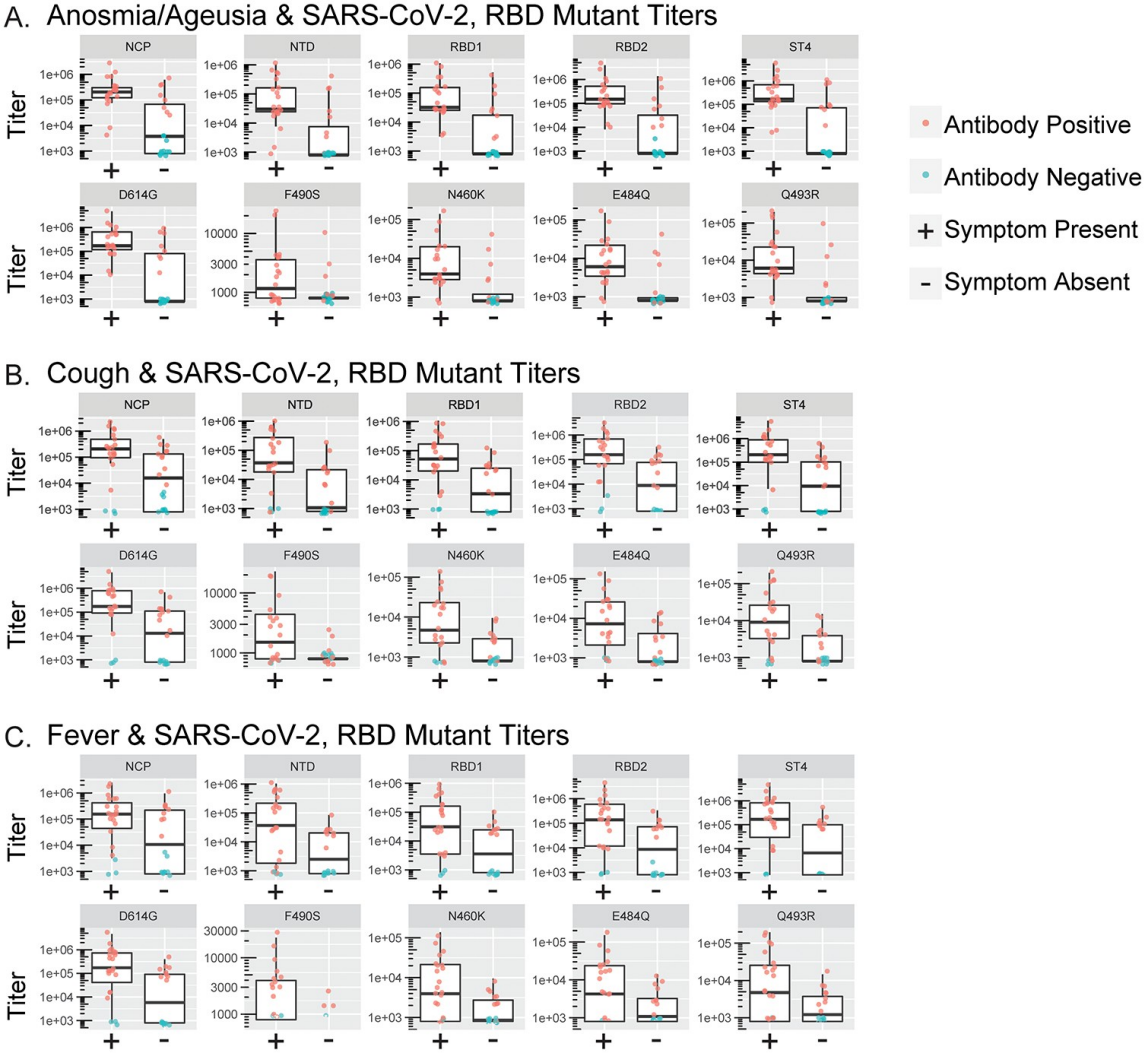
Convalescent plasma antibody titers from pre-vaccinated participants compared to participant reported symptoms. Spike protein antibody titers were analyzed in convalescent plasma samples from pre-vaccinated participants (n = 41). Pre-vaccinated participant titer comparison for SARS-CoV-2 spike protein antigens (NCP, NTD, RBD1, RBD2, and ST4) and RBD mutants (D614G, F490S, N460K, E484Q, Q493R) within participants that reported a presence or absence of anosmia/ageusia (*A*), cough (*B*), and fever (*C*) in symptom questionnaires. AY-Axis ranges reflect the optimized titer signal intensity and distributions based on individual protein antigen and background binding characteristics. Boxes and horizontal bars denote the interquartile range (IQR). The whiskers are equal to the maximum and minimum values below or above the median at 1.5 times the IQR. Antibody positive individuals are labeled in red, and antibody negative individuals are labeled in blue. Pre/pre-vax = pre-vaccinated.

Participants completed symptom questionnaires which included a symptom inventory of fever, chills, rigor, myalgias, headache, sore throat, anosmia and ageusia, cough, dyspnea, wheezing, diarrhea, nausea, and emesis. Symptoms were compared to antibody titers and significant associations were seen with several SARS-CoV-2 antibody titers and anosmia and ageusia, cough, and fever (Fig 2). The summarized results (Table 3) reveal that anosmia and ageusia had significant associations with all titers measured, except for F490S. Cough was similarly significantly associated with all titers measured, except F490S and NCP. Fever was only significantly associated with RBD1, ST4, and D614G. The remaining symptoms (chills, diarrhea, dyspnea, emesis, headache, myalgias, nausea, rigor, sore throat, and wheezing), sex, race, ethnicity, and self-assessed overall disease severity were not significantly associated with any of the SARS-CoV-2 antigens measured.

**Table 3:** Q values for reported symptoms in pre-vaccinated participants.

Antigens/Controls	Antigen Abbreviation	Anosmia/Ageusia N = 21/41 (51%)	Cough N = 21/41 (51%)	Fever N = 23/41 (56%)
**SARS-CoV-2 Proteins**	**NCP**	0.0084*	0.0886	0.1664
	**NTD**	0.0064*	0.0462*	0.0568
	**RBD1**	0.0064*	0.0462*	0.0462*
	**RBD2**	0.0064*	0.0275*	0.0540
	**ST4**	0.0064*	0.0338*	0.0462*
**SARS-CoV-2 RBD Mutant Proteins**	**D614G**	0.0064*	0.0338*	0.0315*
	**F490S**	0.0963	0.0540	0.0945
	**N460K**	0.0064*	0.0462*	0.0886
	**E484Q**	0.0064*	0.0462*	0.1345

### SARS-CoV-2 antibody titers after COVID-19 disease and subsequent COVID-19 vaccination, and impact of participant-reported symptoms

**Fig 3:**
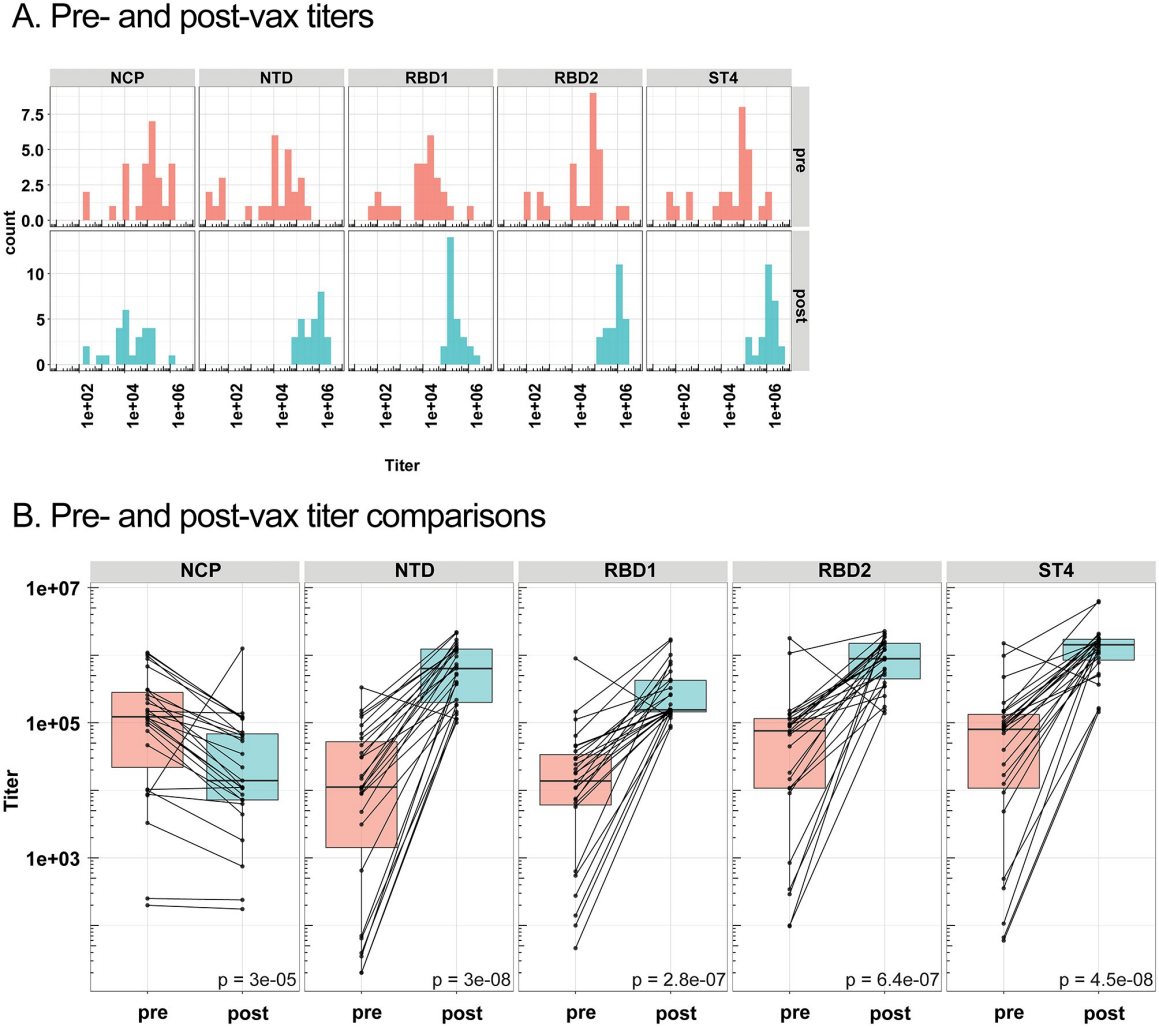
Pre- & post-vaccinated participant sars-cov-2 antibody titer comparisons. *A*, Spike protein antibody titers were analyzed in convalescent plasma samples from pre-vaccinated, participants that returned for the post-vaccinated visit (n = 27, red labeling), and participants from the post-vaccinated visit (n = 27, blue labeling). Median titers for antigens are represented on the x-axis, against the number of participants with median titer represented on the y-axis. If the maximum signal of a titration curve is less than the cut point, then the titer is imputed as 20 (smallest dilution). *B*, Pre- and post-vaccinated participant titer comparison for NCP, NTD, RBD1, RBD2, and ST4. Boxes and horizontal bars denote the interquartile range (IQR). The whiskers are equal to the maximum and minimum titer values below or above the median at 1.5 times the IQR. Statistical significance between groups was determined by non-parametric t-test. The differences were considered statistically significant when p<0.05. Pre/pre-vax = pre-vaccinated, post/post-vax = post vaccination.

Plasma isolated from pre-vaccinated participant blood samples were re-assayed in conjunction with plasma isolated from post-vaccinated participant blood samples (n = 27). Based on relationships identified between spike proteins and COVID-19 symptoms during analysis of pre-vaccinated samples, plasma for all samples were only analyzed for SARS-CoV-2 spike proteins NCP, NTD, RB1, RBD2, and ST4. Titer histograms displayed shifts in post-vaccinated participant spike protein antibody titers (Fig 3A). If the maximum signal of a titration curve is less than the cut point, then the titer is imputed as 20 (smallest dilution). Mean spike protein antibody titers to NTD, RBD1, RBD2, and ST4 were significantly increased in post-vaccinated titers compared to pre-vaccinated titers (Fig 3B). As anticipated, NCP titers were not increased by vaccination.

**Fig 4:**
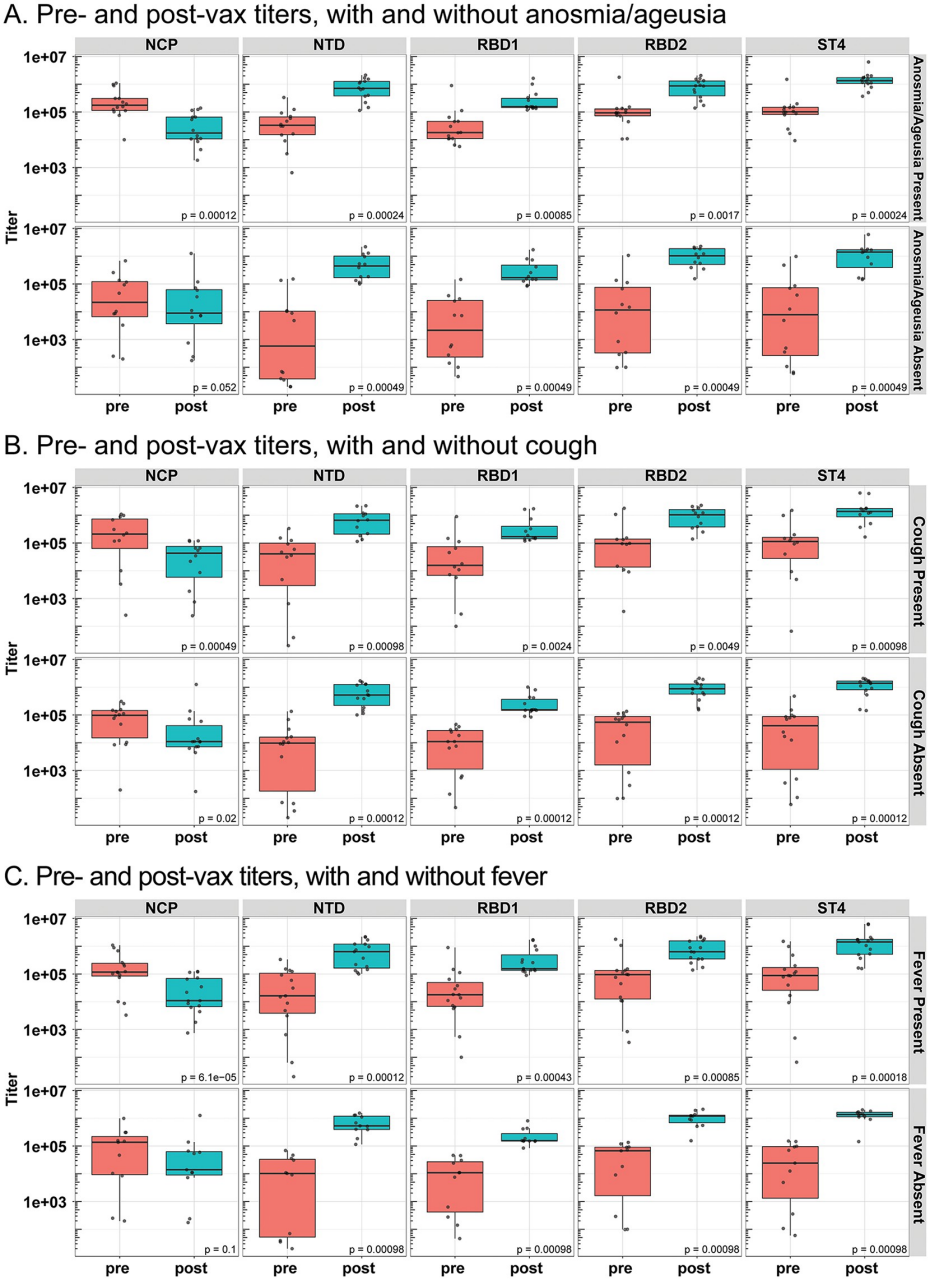
Pre- & post-vaccinated participant SARS-CoV-2 antibody titer comparisons in presence and absence of anosmia/ageusia, cough, and fever. Spike protein antibody titers were analyzed in convalescent plasma samples from pre-vaccinated participants that returned for the post-vaccinated visit (n = 27, red labeling), and participants from the post-vaccinated visit (n = 27, blue labeling). Pre- and post-vaccinated participant titer comparison for NCP, NTD, RBD1, RBD2, and ST4 within participants that reported a presence or absence of anosmia/ageusia (*A*), cough (*B*), and fever (*C*) in symptom questionnaires. Boxes and horizontal bars denote the interquartile range (IQR). The whiskers are equal to the maximum and minimum titer values below or above the median at 1.5 times the IQR. Statistical significance between groups was determined by non-parametric t-test. The differences were considered statistically significant when p<0.05. Pre/pre-vax = pre-vaccinated, post/post-vax = post vaccination.

Pre- and post-vaccinated mean titers were analyzed according to previously reported anosmia and ageusia, cough, and fever, designated as symptom present (Fig 4). Increases in NTD, RBD1, RBD2, and ST4 titers were observed in post-vaccinated participant samples compared to pre-vaccinated participant samples, regardless of the presence or absence of previously reported anosmia and ageusia (Fig 4A), cough (Fig 4B), or fever (Fig 4C) presented during the initial SARS-CoV-2 infection. In contrast, NCP titers were reduced in titers in post-vaccinated samples compared to titers from pre-vaccinated samples within anosmia and ageusia, cough, and fever present analysis. Statistical comparisons between pre-vaccinated titer symptom present and pre-vaccinated titer symptom absent groups were significant for NCP, NTD, RBD1, RBD2, and ST4 within anosmia and ageusia, cough, and fever-presenting groups (Table 4, S2 Fig) and confirmed initial results from phase 1 where a different dilution was used. In all spike protein antibody titers, symptom absent participants had significantly reduced titers compared to symptom present participants. Comparisons between post-vaccinated titers in symptom present groups and post-vaccinated titers in symptom absent groups were not statistically significant for any of the SARS-CoV-2 protein antigens tested (Table 4, S3 Fig).

**Table 4:** P-values for symptom present and absent comparisons within pre-vaccinated or post-vaccinated titers and ACE2 IC50.

	NCP	NTD	RBD1	RBD2	ST4	ACE2 IC50
**Pre-vaccinated symptom present and absent**						
Fever	0.013*	0.0065*	0.0022*	0.0036*	0.002*	0.0072*
Cough	0.0016*	0.0012*	0.0024*	0.00023*	0.00093*	0.00055*
Anosmia/Ageusia	0.0001*	0.00017*	0.00012*	0.000092*	0.0001*	0.00065*
**Post-vaccinated symptom present and absent**						
Fever	0.72	0.80	0.92	0.44	0.96	0.68
Cough	0.56	0.94	0.70	0.94	0.70	0.49
Anosmia/Ageusia	0.35	0.27	0.94	0.46	0.56	0.94

### ACE2 neutralization after COVID-19 disease and subsequent COVID-19 vaccination, and impact of participant-reported symptoms

**Fig 5:**
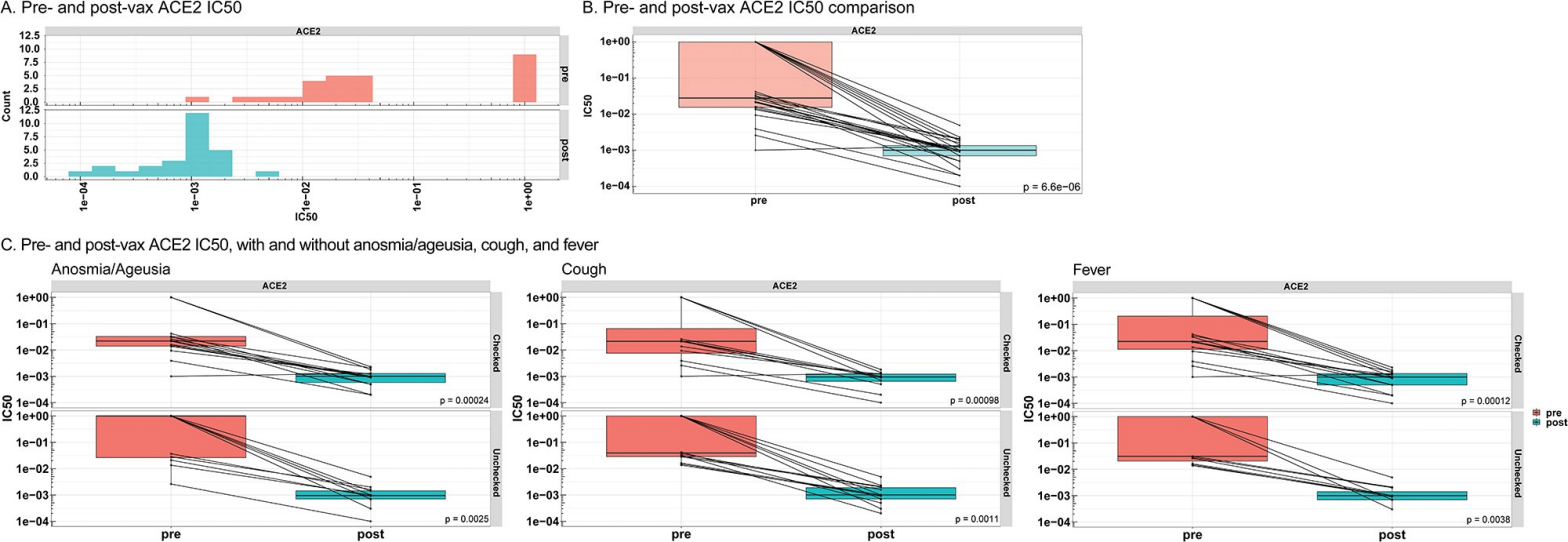
Pre- & post-vaccinated participant ACE2 neutralization comparisons in presence and absence of anosmia/ageusia, cough, and fever. *A*, ACE2 IC50 was analyzed in convalescent plasma samples from pre-vaccinated participants that returned for the post-vaccinated visit (n = 27, red labeling), and participants from the post-vaccinated visit (n = 27, blue labeling). *B*, Pre- and post-vaccinated IC50 comparison for ACE2. *C*, Pre- and post-vaccinated ACE2 IC50 comparisons within symptom-present and symptom-absent participants for anosmia/ageusia, cough, and fever from symptom questionnaires. Boxes and horizontal bars denote the interquartile range (IQR). The whiskers are equal to the maximum and minimum titer values below or above the median at 1.5 times the IQR. Statistical significance between groups was determined by non-parametric t-test. The differences were considered statistically significant when p<0.05. Pre/pre-vax = pre-vaccinated, post/post-vax = post vaccination, IC50 = half-maximal inhibitory concentration, ACE2 = angiotensin converting enzyme 2.

ACE2 neutralization was assessed in pre- and post-vaccinated participant plasma samples. The IC50 was reported as a measure of antibody neutralization efficacy, with lower values indicating higher neutralization efficacy. Pre-vaccinated participant samples from symptom absent participants displayed significantly greater ACE2 IC50 compared to participants that presented anosmia and ageusia, cough, and fever (Table 4, S4 Fig), in line with lower antibody titers observed in the symptom absent participants. The ACE2 IC50 significantly decreased in samples collected post-vaccination and suggests an increase in antibody potency for ACE2 inhibition (Fig 5A and 5B). Decreases in ACE2 IC50 were consistent in anosmia and ageusia, cough, and fever present and absent groups (Fig 5C). Statistical comparisons between post-vaccinated ACE2 IC50 symptom present and post-vaccinated ACE2 IC50 symptom absent samples were not significant (Table 4, S5 Fig).

## Discussion

### Summary

During the initial wave of the pandemic, US health system employees representative of diverse age, gender, and race/ethnicity backgrounds exhibited clearly detectable antibody titers forSARS-CoV-2 proteins and RBD mutant proteins, 8–10 weeks after onset of COVID-19 infection. Antibody titers to ST4, RBD1, and RBD mutant D614G were significantly associated with participant-reported anosmia and ageusia, cough, and fever, suggesting that severity of infection influences the magnitude of the humoral immune response. Prior to a 2-dose series of mRNA BNT162b2 COVID-19 vaccine, spike protein antibody titers and ACE2 neutralization were significantly higher in participants presenting with these symptoms. Vaccination comparatively increased antibody titers for NTD, RBD1, RBD2 and ST4, and increased ACE2 neutralization regardless of the original presence of anosmia and ageusia, cough, or fever. These results reveal previously unreported relationships between clinical COVID-19 symptomology and SARS-CoV-2 antibody titer and neutralization responses after COVID-19 disease and after a 2-dose series mRNA vaccination.

### Pre-vaccinated participant samples

Spike protein antibody titers detectable in 71% of recovered participants suggests a humoral immune response to SARS-CoV-2, 8 to 10 weeks from COVID-19 infection. These results align with reports of stable spike IgG after COVID-19 disease, suggested to remain longitudinally stable over 6 months (or 24 weeks) after SARS-CoV-2 symptom onset [9]. SARS-CoV-2 spike protein seropositivity is associated with reduced risk of reinfection after SARS-CoV-2 infection [18, 19] and suggests protection to reinfection among the 71% of spike titer-responsive participants within this post-infection timeframe. There were no reports of reinfection in any of the participants, as assessed with the patient questionnaires at the phase 1 and phase 2 visits.

The remaining 29% of participants that did not exhibit detectable antibody titers to the SARS-CoV-2 spike protein antigens reported fewer symptoms, including no cases of anosmia and ageusia, and suggests mild illness. Previous reports demonstrate a similar 23.3% of individuals that did not exhibit detectable IgG antibody titers to SARS-CoV-2 spike protein in hospitalized patients within 3–4 weeks of COVID-19 symptom onset [15]. Similarly, in a cohort of primarily non-hospitalized (93%) patients analyzed during the initial wave of SARS-CoV-2 in New York, 30% of plasma anti-spike IgG samples were less than two standard deviations above controls approximately 39 days (or 6 weeks) after symptom onset [16]. The lack of detectable antibody responses reported up to 6 weeks after COVID-19 disease could be related to individual disease severity and subsequent immunity, as both are correlated with the SARS-CoV-2 spike IgG titer response [17]. However, studies that identified populations with undetectable spike protein IgG did not provide additional analyses relating disease severity or symptom presentation. Alternatively, undetectable antibody titers could be a consequence of rapid decreases in spike antibody titers, based on evidence that spike antibody titers can rapidly wane within 50 days (or 7 weeks) of COVID-19 symptom onset [18].

Although unvaccinated cohort samples were not compared to the vaccinated cohort in phase 2 blood draw samples, the studies mentioned [16–18] and that reported by Townsend et al., 2021 [19] estimate natural immunity antibody decline and the potential for reinfection to occur 3 months to 5.1 years after infection during endemic conditions [24]. The large range suggests individual differences in the natural immune response, which could be a result of the primary infection disease severity and/or symptom presentation.

D614G transitioned to the dominant variant and represented 78% of 12,194 sequences investigated worldwide [20] during the start of pre-vaccinated participant enrollment May-July 2020 and following initial virus exposure and infection February-May 2020. Although the D614G RBD mutant in pre-vaccinated participants had the highest titer values among RBD mutant antigens measured, titer magnitude is not comparable among antigens due to differences in antigen sizes that result in higher or lower bound antibody according to the antigen probe.

ACE2 neutralization within the pre-vaccinated participants was detectable but was significantly greater in participants that expressed anosmia and ageusia, cough, or fever compared to participants without specific symptoms. Significant association between COVID-19 symptoms and ACE2 neutralization suggests an opportunity for clinical utility between symptom presentation and SARS-CoV-2 immunity after SARS-CoV-2 infection.

### Post-vaccinated participant samples

In contrast to spike protein antibody titers in pre-vaccinated participants, all post-vaccinated participants exhibited detectable antibody titers to spike proteins and NTD, RBD1 and 2, and ST4 titers increased compared to pre-vaccinated titers. Others have reported comparable increases in spike protein IgG after vaccination in individuals previously infected with SARS-CoV-2, while anti-NCP IgG remains unchanged [21, 22]. Neutralizing activity can also be potentiated by SARS-CoV-2 mRNA vaccination. The ACE2 neutralization results in this study suggest potentiated immunity to SARS-CoV-2 after vaccination compared to pre-vaccinated immunity to SARS-CoV-2. These results may support a distinct efficiency in the vaccine-induced IgG response and neutralization compared to the COVID-19-induced IgG response. Distinct from pre-vaccination status, previous anosmia and ageusia, fever, and cough did not impact spike protein antibody titers or ACE2 IC50 after SARS-CoV-2 vaccination. The lack of symptom impact on vaccine-induced titers and ACE2 IC50 suggests that discrete symptom profiles associated with illness severity are not predictive in a mechanism of memory B cell immune response. Specifically, a B cell immune response mediated by the intensity and duration of previous COVID-19 infection may not contribute to the evidently robust neutralizing activity after mRNA SARS-CoV-2 immunization [23, 24].

Although all post-vaccinated participant samples displayed higher ACE2 IC50 than in pre-vaccinated participant samples, values splayed into high and low ACE2 IC50 concentrations in samples from pre-vaccinated participants. Nine participants within the pre-vaccinated cohort showed high IC50, indicative of low ACE2 neutralization. These samples had the lowest titers for ST4, RBD2, RBD1, NTD and NCP. None of these participants experienced fever, only one experienced anosmia/ageusia, and three experienced cough, and eight of the nine participants with high ACE2 IC50 in pre-vaccinated samples experienced minor or asymptomatic illness severity. These results further suggest a direct relationship between ACE2 neutralization, titer response, and symptom presentation and severity in response to COVID-19 infection.

The BNT162b2 COVID-19 vaccine targeted sequence does not target the D614G mutant and was therefore rationale for exclusion of D614G during the second analysis of pre- and post-vaccinated samples together. However, spike protein variants d69-70 and E484Q were included in the second analysis with pre- and post-vaccinated participant analysis (S6 Fig). Inclusion was based on residues of interest on the omicron evolving strain at the time of analysis with implications for diagnostic testing performance and immune escape.

### Study limitations

Limitations of this study include the small sample size and incomplete (66%) retention of participants that returned for a post-vaccination visit. Also, viral load cycle threshold values were not available from confirmed PCR positive tests during symptomatic illness, which restricted the ability to correlate viral load levels with antibody titers [25]. In addition, we did not study antibody titers after the first vaccination dose.

## Conclusions

These results support clinically meaningful relationships between antibodies to SARS-CoV-2 spike protein and COVID-19 symptom presentation of anosmia and ageusia, cough, and fever. Results of this study may be useful to inform clinicians of the potential duration of post-infection seroprotective immunity characterized by baseline reported COVID-19 illness symptoms, as well as the insignificance of previous SARS-CoV-2 symptom presentation on post-vaccine immunity.

## Supporting information

S1 Fig Study enrollment and trial design. (TIF)

S2 Fig Symptom impact on SARS-CoV-2 antigen titers in pre-vaccinated participants. Comparisons were made in pre-vaccinated participant samples (n = 41) between median titers for SARS-CoV-2 antigens (NCP, NTD, RBD1, RBD2, ST4, d69-70 and E848Q) and anosmia and ageusia, cough, and fever presence and absence. All titers were significantly higher in samples from symptom present compared to symptom absent participants (non-parametric t-test p<0.05).
(TIF)

S3 Fig Symptom impact on SARS-CoV-2 antigen titers in post-vaccinated participants. Comparisons were made in post-vaccinated participant samples (n = 27) between median titers for SARS-CoV-2 antigens (NCP, NTD, RBD1, RBD2, ST4, d69-70 and E848Q) and anosmia and ageusia, cough, and fever presence and absence. No titers were significantly different in samples from symptom present compared to symptom absent participants (non-parametric t-test p<0.05).
(TIF)

S4 Fig Symptom impact on ACE2 IC50 in pre-vaccinated participants. Comparisons were made in pre-vaccinated participant samples (n = 41) between median ACE2 IC50 and anosmia and ageusia, cough, and fever presence and absence. ACE2 neutralization was significantly increased in samples from symptom present vs symptom absent patients (non-parametric t-test p<0.05).
(TIF)

S5 Fig Symptom impact on ACE2 IC50 in post-vaccinated participants. Comparisons were made in post-vaccinated participant samples (n = 27) between median ACE2 IC50 and anosmia/ageusia, cough, and fever presence and absence. ACE2 neutralization was not significantly different in samples from symptom present compared to symptom absent participants (non-parametric t-test p<0.05).
(TIF)

S6 Fig Pre- & post-vaccinated participant SARS-CoV-2 d69-70 NTD and E484Q antibody titer comparisons and symptom impact on titers. A, d69-70 NTD and E484Q titers were analyzed in convalescent plasma samples from pre-vaccinated participant samples that returned for the post-vaccinated visit (n = 27, red labeling), and post-vaccinated participant samples (n = 27, blue labeling). Antigen titers are represented on the x-axis, against the number of participants represented on the y-axis. If the maximum signal of a titration curve is less than the cut point, then the titer is imputed as 20 (smallest dilution). B, Pre- and post-vaccinated participant titer comparison for d69-70 NTD and E484Q, and C, according to symptom presence. Boxes and horizontal bars denote the interquartile range (IQR). The whiskers are equal to the maximum and minimum titer values below or above the median at 1.5 times the IQR. Statistical significance between groups was determined by non-parametric t-test. The differences were considered statistically significant when p<0.05. Pre/pre-vax = pre-vaccinated, post/post-vax = post vaccination.
(DOCX)

S1 File Phase 1 online survey. (DOCX)

S2 File Phase 2 online survey. (DOCX)
