# Supplementary figures and images for: COVID-19 symptom relationship to antibody response and ACE2 neutralization in recovered health systems employees before and after mRNA BNT162b2 COVID-19 vaccine
Source: PLoS One. 2022 Sep 9;17(9):e0273323. doi: 10.1371/journal.pone.0273323 (PMC9462709; doi:10.1371/journal.pone.0273323)

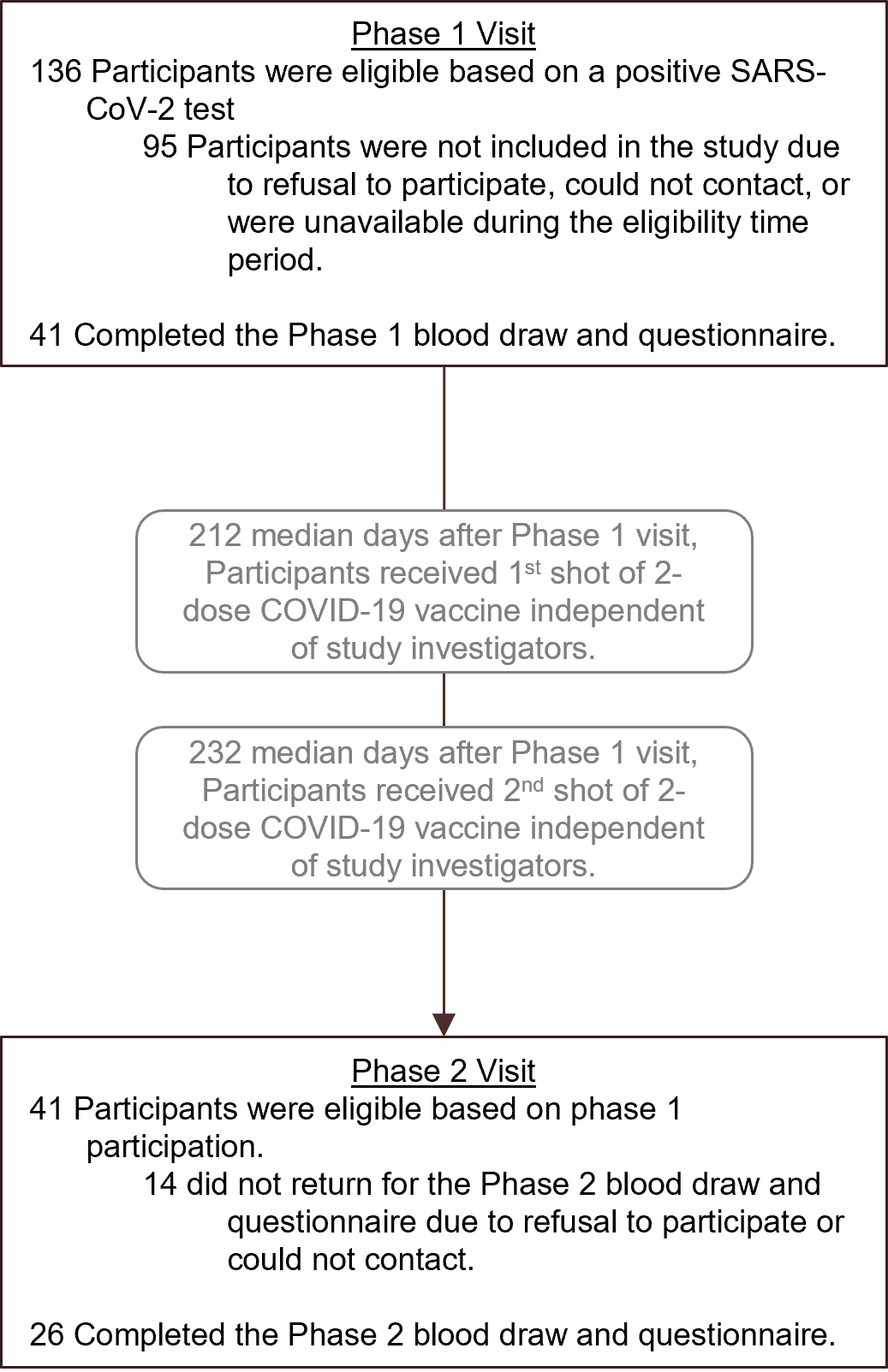

Supplement: S1 Fig — (TIF) [file pone.0273323.s001.tif]
