# Supplementary material for: COVID-19 symptom relationship to antibody response and ACE2 neutralization in recovered health systems employees before and after mRNA BNT162b2 COVID-19 vaccine
Source: PLoS One. 2022 Sep 9;17(9):e0273323. doi: 10.1371/journal.pone.0273323 (PMC9462709; doi:10.1371/journal.pone.0273323)
Supplement: S6 Fig — A, d69-70 NTD and E484Q titers were analyzed in convalescent plasma samples from pre-vaccinated participant samples that returned for the post-vaccinated visit (n = 27, red labeling), and post-vaccinated participant samples (n = 27, blue labeling). Antigen titers are represented on the x-axis, against the number of participants represented on the y-axis. If the maximum signal of a titration curve is less than the cut point, then the titer is imputed as 20 (smallest dilution). B, Pre- and post-vaccinated participant titer comparison for d69-70 NTD and E484Q, and C, according to symptom presence. Boxes and horizontal bars denote the interquartile range (IQR). The whiskers are equal to the maximum and minimum titer values below or above the median at 1.5 times the IQR. Statistical significance between groups was determined by non-parametric t-test. The differences were considered statistically significant when p<0.05. Pre/pre-vax = pre-vaccinated, post/post-vax = post vaccination. (DOCX) [file pone.0273323.s006.docx]

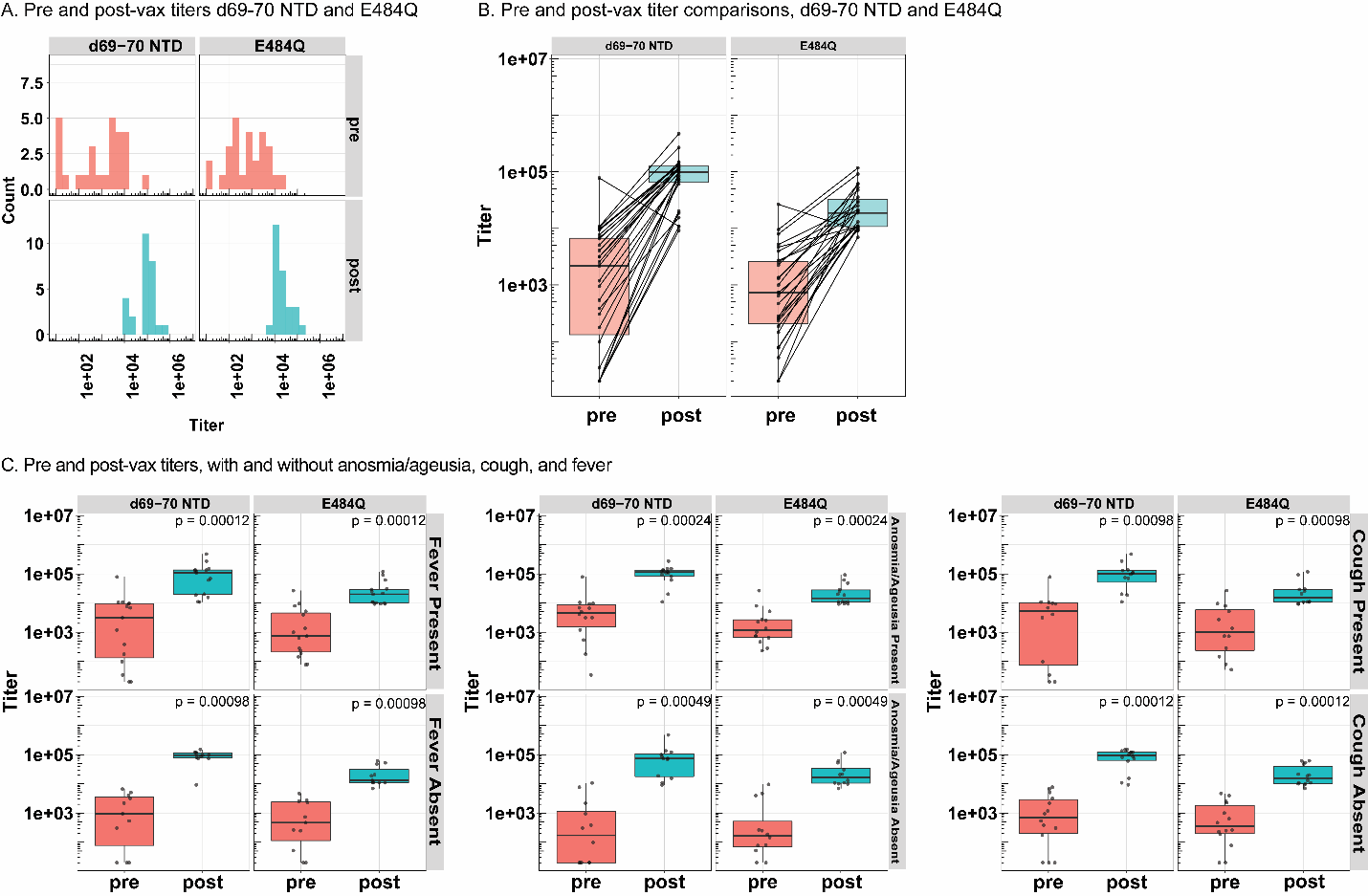


**S6. Pre- & Post-Vaccinated Participant SARS-CoV-2 d69-70 NTD and E484Q Antibody Titer Comparisons and Symptom Impact on Titers**

*A*, d69-70 NTD and E484Q titers were analyzed in convalescent plasma samples from pre-vaccinated participant samples that returned for the post-vaccinated visit (n=27, red labeling), and post-vaccinated participant samples (n=27, blue labeling). Antigen titers are represented on the x-axis, against the number of participants represented on the y-axis. If the maximum signal of a titration curve is less than the cut point, then the titer is imputed as 20 (smallest dilution). *B*, Pre- and post-vaccinated participant titer comparison for d69-70 NTD and E484Q, and C, according to symptom presence. Boxes and horizontal bars denote the interquartile range (IQR). The whiskers are equal to the maximum and minimum titer values below or above the median at 1.5 times the IQR. Statistical significance between groups was determined by non-parametric t-test. The differences were considered statistically significant when p<0.05. Pre/pre-vax= pre-vaccinated, post/post-vax= post vaccination.
