# Supplementary material for: COVID-19 symptom relationship to antibody response and ACE2 neutralization in recovered health systems employees before and after mRNA BNT162b2 COVID-19 vaccine
Source: PLoS One. 2022 Sep 9;17(9):e0273323. doi: 10.1371/journal.pone.0273323 (PMC9462709; doi:10.1371/journal.pone.0273323)
Supplement: S1 File — (DOCX) [file pone.0273323.s007.docx]

**S7. Phase 1 Online Survey**

Please complete the survey below to the best of your ability. Questions or clarifications may be directed to the research staff assisting you today. If you have any questions during the course of the survey please do not hesitate to ask.

Thank you for your participation! Thank you for being a part of the ACCELERATED Study. Please answer all questions below to the best of your ability. If you have any questions during the course of the survey please do not hesitate to ask.

How old are you?

What is your gender?

- Male
- Female
- Transgender Male
- Transgender Female
- Other

If you selected “other” under gender, please list it here.

Do you identify as Hispanic, Latino or Spanish?

- Yes
- No

How do you describe your ethnic background?

- Black/African American
- White
- Asian
- American Indian or Alaska Native
- Native Hawaiian and Public Islanders
- Multi-Racial/Bi-Racial
- Other

What is you home zip code?

What was the date of the onset of your COVID-19 symptoms? (Best estimate)

What was the date of your COVID-19 test?

Were you diagnosed with pneumonia with a chest xray?

- Yes
- No

What symptoms did you experience? Check all that apply.

- Fever
- Chills
- Repreated shaking with chills
- Muscle pain
- Headache
- Sore throat
- New loss of taste or smell
- Cough
- Shortness of breath
- Wheezing
- Diarrhea
- Nausea
- Vomiting
- Other
- None

What other symptoms did you have?

What was the duration of your fever?

- 1-3 days
- 4-7 days
- 1-2 weeks
- 2-3 weeks
- 4+ weeks

What was the duration of your chills?

- 1-3 days
- 4-7 days
- 1-2 weeks
- 2-3 weeks
- 4+ weeks

What was the duration of your repeated shaking with chills?

- 1-3 days
- 4-7 days
- 1-2 weeks
- 2-3 weeks
- 4+ weeks

What was the duration of your muscle pain?

- 1-3 days
- 4-7 days
- 1-2 weeks
- 2-3 weeks
- 4+ weeks

What was the duration of your headaches?

- 1-3 days
- 4-7 days
- 1-2 weeks
- 2-3 weeks
- 4+ weeks

What was the duration of your sore throat?

- 1-3 days
- 4-7 days
- 1-2 weeks
- 2-3 weeks
- 4+ weeks

What was the duration of your new loss of taste or smell?

- 1-3 days
- 4-7 days
- 1-2 weeks
- 2-3 weeks
- 4+ weeks

What was the duration of your cough?

- 1-3 days
- 4-7 days
- 1-2 weeks
- 2-3 weeks
- 4+ weeks

What was the duration of your shortness of breath?

- 1-3 days
- 4-7 days
- 1-2 weeks
- 2-3 weeks
- 4+ weeks

What was the duration of your wheezing?

- 1-3 days
- 4-7 days
- 1-2 weeks
- 2-3 weeks
- 4+ weeks

What was the duration of your diarrhea?

- 1-3 days
- 4-7 days
- 1-2 weeks
- 2-3 weeks
- 4+ weeks

What was the duration of your nausea?

- 1-3 days
- 4-7 days
- 1-2 weeks
- 2-3 weeks
- 4+ weeks

What was the duration of your vomiting?

- 1-3 days
- 4-7 days
- 1-2 weeks
- 2-3 weeks
- 4+ weeks

What was the duration of your other symptoms?

- 1-3 days
- 4-7 days
- 1-2 weeks
- 2-3 weeks
- 4+ weeks

Did you experience any complications from COVID-19?

- None or very minor
- Moderate-less important than the diagnosis
- Major
- Catastrophic

During your illness, how would you rate your level of dependency?

- Low
- Moderate
- Major
- Extreme

What procedures did you undergo when you were ill?

- Noninvasive diagnostic or minor therapeutic
- Therapeutic or invasive diagnostic
- Nonemergency life support
- Emergency life support

What therapy or treatment did you receive while you were ill (including prescription and over the counter medication)?

What was your response to therapy/treatment?

- Prompt
- Moderate delay
- Serious delay
- No response

Please rate the severity of your illness.

- 1-asymptomatic
- 2-minor
- 3-major
- 4 catastrophic

How many weeks after the onset of symptoms did you recover?

Please list any pre-existing medical conditions.
