# Supplementary material for: COVID-19 symptom relationship to antibody response and ACE2 neutralization in recovered health systems employees before and after mRNA BNT162b2 COVID-19 vaccine
Source: PLoS One. 2022 Sep 9;17(9):e0273323. doi: 10.1371/journal.pone.0273323 (PMC9462709; doi:10.1371/journal.pone.0273323)
Supplement: S2 File — (DOCX) [file pone.0273323.s008.docx]

**S8. Phase 2 Online Survey**

Please complete the survey below to the best of your ability. Questions or clarifications may be directed to the research staff assisting you today. If you have any questions during the course of the survey please do not hesitate to ask.

Thank you for your participation! Thank you for being a part of the ACCELERATED Study.

Have there been any significant changes to your known comorbidities? Yes or No

If yes, what were they? _________________________________________________________________

What was the last day of your COVID-19 symptoms? __________________________________________

Did you experience any complications from COVID-19?

- None or very minor
- Moderate (less important than the diagnosis)
- Major
- Catastrophic (hospitialization)

Have you had any persistent symptoms from COVID-19? Yes or No

If yes, what were they? _________________________________________________________________

Did you experience any post-vaccination symptoms after the first dose?

If yes, what post-vaccination symptoms did you experience after your first dose? Check all that apply.

- Fever
- Chills
- Repeated shaking with chills
- Muscle pain
- Headache
- Shortness of breath
- Diarrhea
- Nausea
- Vomiting
- Anaphylactic reaction
- Other
- None

If you selected “other,” what symptoms did you have? ________________________________________

What was the duration of your symptoms after the first dose?

- 1-3 days
- 4-7 days
- 1-2 weeks
- 2-3 weeks
- 4+ weeks

Did you experience any post-vaccination symptoms after the second dose?

If yes, what post-vaccination symptoms did you experience after your second dose? Check all that apply.

- Fever
- Chills
- Repeated shaking with chills
- Muscle pain
- Headache
- Shortness of breath
- Diarrhea
- Nausea
- Vomiting
- Anaphylactic reaction
- Other
- None

If you selected “other,” what symptoms did you have? ________________________________________

What was the duration of your symptoms after the second dose?

- 1-3 days
- 4-7 days
- 1-2 weeks
- 2-3 weeks
- 4+ weeks

Thank you for your time and participation!
